# Pediatric joint hypermobility: a diagnostic framework and narrative review

**DOI:** 10.1186/s13023-023-02717-2

**Published:** 2023-05-04

**Authors:** Louise Jane Tofts, Jane Simmonds, Sarah B. Schwartz, Roberto M. Richheimer, Constance O’Connor, Ellen Elias, Raoul Engelbert, Katie Cleary, Brad T. Tinkle, Antonie D. Kline, Alan J. Hakim, Marion A. J. van Rossum, Verity Pacey

**Affiliations:** 1 Macquarie University, 75 Talavera Rd, Sydney, NSW 2109 Australia; 2 Great Ormond Street Institute of Child Health, University College London, London, UK; 3 London Hypermobility Unit, Central Health Physiotherapy, London, UK; 4 Hospital for Sick Children, 555 University Ave, Toronto, ON M5G 1X8 Canada; 5 Centro Médico ABC, Carlos Graef Fernández 154-1A, Col. Tlaxala, Alc. Cuajimalpa de Morelos, 05300 Mexico City, CDMX Mexico; 6 University of Colorado School of Medicine, Denver, USA; 7 Ehlers-Danlos Center for Excellence and Special Care Clinic, Children’s Hospital Colorado Special Care Clinic, Aurora, CO USA; 8 Department of Rehabilitation Medicine, Amsterdam University Medical Center (AMC), Meiberg Dreef 9, 1105 AZ Amsterdam, The Netherlands; 9 Ocean Kids Physio, Unit 1/2-8 Peninsula Blvd, Seaford, VIC 3198 Australia; 10 Peyton Manning Children’s Hospital, 8402 Harcourt Rd, Ste 300, Indianapolis, IN 46260 USA; 11 Greater Baltimore Medical Center, Harvey Institute for Human Genetics, 6701 N. Charles St., Suite 2326, Baltimore, MD 21204 USA; 12 The Harley Street Clinic, HCA Healthcare, 16 Devonshire Street, London, UK; 13 Emma Children’s Hospital Academic Medical Centre, Amsterdam, The Netherlands

**Keywords:** Child, Adolescent, Joint hypermobility, Ehlers–Danlos syndrome

## Abstract

**Background:**

Hypermobile Ehlers–Danlos syndrome (hEDS) and hypermobility spectrum disorders (HSD) are debilitating conditions. Diagnosis is currently clinical in the absence of biomarkers, and criteria developed for adults are difficult to use in children and biologically immature adolescents. Generalized joint hypermobility (GJH) is a prerequisite for hEDS and generalized HSD. Current literature identifies a large proportion of children as hypermobile using a Beighton score ≥ 4 or 5/9, the cut off for GJH in adults. Other phenotypic features from the 2017 hEDS criteria can arise over time. Finally, many comorbidities described in hEDS/HSD are also seen in the general pediatric and adolescent population. Therefore, pediatric specific criteria are needed. The Paediatric Working Group of the International Consortium on EDS and HSD has developed a pediatric diagnostic framework presented here. The work was informed by a review of the published evidence.

**Observations:**

The framework has 4 components, GJH, skin and tissue abnormalities, musculoskeletal complications, and core comorbidities. A Beighton score of ≥ 6/9 best identifies children with GJH at 2 standard deviations above average, based on published general population data. Skin and soft tissue changes include soft skin, stretchy skin, atrophic scars, stretch marks, piezogenic papules, and recurrent hernias. Two symptomatic groups were agreed: musculoskeletal and systemic. Emerging comorbid relationships are discussed. The framework generates 8 subgroups, 4 pediatric GJH, and 4 pediatric generalized hypermobility spectrum disorders. hEDS is reserved for biologically mature adolescents who meet the 2017 criteria, which also covers even rarer types of Ehlers–Danlos syndrome at any age.

**Conclusions:**

This framework allows hypermobile children to be categorized into a group describing their phenotypic and symptomatic presentation. It clarifies the recommendation that comorbidities should be defined using their current internationally accepted frameworks. This provides a foundation for improving clinical care and research quality in this population.

## Introduction

The 2017 criteria for hypermobile Ehlers–Danlos syndrome (hEDS) [1] and hypermobility spectrum disorders (HSD) [2] were established based on expert consensus and evidence from adult studies. A diagnosis of hEDS requires the presence of generalized joint hypermobility (GJH), with at least two of: 5+/12 of a set of phenotypic features of mild skin and tissue fragility and a marfanoid habitus; a first-degree relative meeting the criteria; and at least one of daily musculoskeletal pain in 2 or more limbs over 3 months, chronic widespread pain, or joint dislocations or instability [1]. HSD is currently described in individuals with any of generalized, peripheral (hands and/or feet), localized (any single joint) or historic joint hypermobility and associated symptoms in the absence of other cause/diagnosis [2]. Biologically mature adolescents can be diagnosed using these criteria, but they are difficult to use in children and biologically immature adolescents (herewith children), who have not yet developed a stable phenotype.

Children have high levels of joint hypermobility (herewith hypermobility) [3, 4] making it difficult to distinguish those with a normal physical trait from those with an underlying disorder [5]. Hypermobility decreases during pediatric years, probably later in adolescent females than males. Skin and soft tissue features may develop with time post growth (stretch marks) or injury (scarring), and musculoskeletal complications and comorbidities can occur in any child [6].

We propose that children should not be assessed with the 2017 criteria or diagnosed with hEDS until they have reached biological maturity, so a pediatric specific framework was developed. The diagnoses are fluid, allowing children with GJH to be reclassified over time as: typical if GJH resolves, asymptomatic GJH as symptoms improve, or pediatric generalized HSD (pgHSD) upon presentation of new signs and symptoms. An accurate pgHSD diagnosis provides the foundation for appropriate current treatment and support, but not a lifelong diagnosis which may result in over medicalization and potential harms. The framework supports identifying those with hypermobility as a physical trait, which is relatively common, those with musculoskeletal issues related to their hypermobility, and those who may develop the rarer hypermobile Ehlers–Danlos syndrome as they mature.

This framework will support targeted genetic testing, which currently has an 11.5% yield [7], and improved consistency when researching epidemiology, symptom evolution, complications, and interventions. 

## Methods

The Paediatric Working Group and representatives of the hEDS/HSD Working Group of the International Consortium on EDS and HSD [8] met online throughout 2020–22. The group comprised the authors, with representation from Europe, The Americas, and Australasia, and from medical, nursing, and physical therapy clinical and academic backgrounds.

Collectively over the last 5 years the group has seen over 5000 pediatric patients for diagnostic evaluation and contributed substantially to the peer-reviewed literature on hypermobility-related epidemiology, disorders, and treatment.

Using a codesign methodology [9], a series of meetings were established to define, design, and refine the framework. Initially, evidence from the literature was reviewed although limited because of varying definitions of GJH and diagnostic criteria, precluding clinically meaningful systematic reviews [10]. Studies of children alone, and both children and adults were included and were not assessed for quality due to the wide variety of populations studied, reporting methods, and study designs.

The evidence base was combined with real-world experience of experts in the group to draft the initial pediatric framework, which was presented at The British Society of Rheumatology Case-based Conference, October 2021; The International Society of Paediatric Pain, 13th International Symposium on Paediatric Pain, March 2022; and The Ehlers–Danlos Society Scientific Symposium, Rome, September 2022. Consultation at these meetings informed a revised framework which was then updated with a final literature review. Stakeholder consultation was sought from the Medical and Scientific Board of the Ehlers–Danlos Society [11] and community stakeholder consultation through the ‘Action for HSD and hEDS Accurate Diagnosis’ (AHEAD) coalition, a group of organizations involved in the welfare of children and young people with HSD/EDS, and their families [12].

## Discussion/observations

**Fig. 1:**
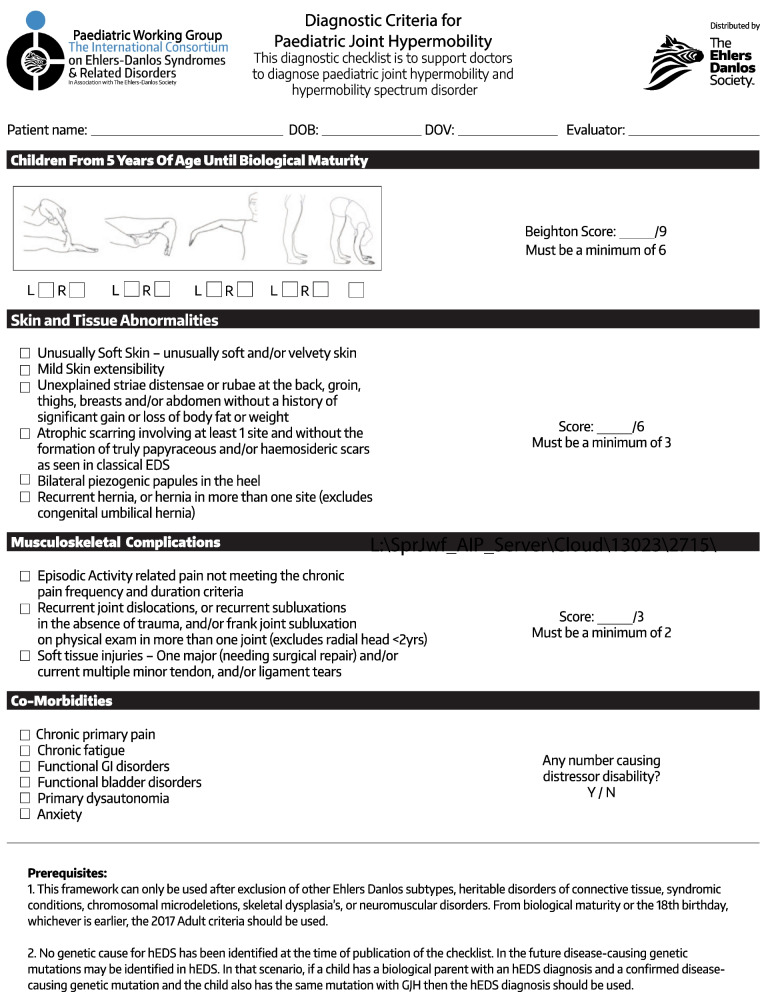
A diagnostic checklist for pediatric joint hypermobility and hypermobilty spectrum disorder

The diagnostic framework is presented in Table 1, with the components and decision making discussed below. The framework is intended to be fluid, so that each child can change subtype as symptoms and joint mobility change. The framework has four main categories, with or without skin involvement which then yields eight in total. An asymptomatic category, with GJH only, is included for young family members in a pedigree, and observational and interventional research. GJH with core comorbidities was included to describe patients without significant MSK symptoms but with comorbidities. Hypermobility spectrum disorder is reserved for the combination of GJH and musculoskeletal symptoms, with the systemic subtype including those with both musculoskeletal symptoms and comorbidities. This can only be used after exclusion of other Ehlers–Danlos syndrome types, heritable disorders of connective tissue, syndromic conditions, chromosomal microdeletions, skeletal dysplasia, or neuromuscular disorders. This framework would not exclude the abovementioned diagnoses, and individual patients would need to be assessed for risk of alternate diagnosis and investigated appropriately, including, if indicated, next-generation sequencing (NGS) panel-based testing. Currently, we do not recommend genetic testing on all children with GJH, or HSD or hEDS. Medical specialists should remain guided by their clinical reasoning and the healthcare setting in which they practice when deciding if genetic testing is required. Figure 1 is a proposed clinic practice checklist applicable to the diagnostic framework and designed to complement the 2017 International Criteria.

**Table 1:** Diagnostic framework for pediatric joint hypermobility in the presence of skin abnormalities, musculoskeletal complications, and/or core comorbid conditions

	Generalized joint hypermobility	Skin and tissue abnormalities	Musculoskeletal complications	Core comorbidities
*Asymptomatic*				
Pediatric generalized joint hypermobility	Present	Absent	Absent	Absent
Pediatric generalized joint hypermobility with skin involvement	Present	Present	Absent	Absent
*Symptomatic conditions*				
Pediatric generalized joint hypermobility with core comorbidities	Present	Absent	Absent	Present
Pediatric generalized joint hypermobility with core comorbidities and with skin involvement	Present	Present	Absent	Present
Pediatric hypermobility spectrum disorder, musculoskeletal subtype	Present	Absent	Present	Absent
Pediatric hypermobility spectrum disorder, musculoskeletal subtype with skin involvement	Present	Present	Present	Absent
Pediatric hypermobility spectrum disorder, systemic subtype	Present	Absent	Present	Present
Pediatric hypermobility spectrum disorder, systemic subtype with skin involvement	Present	Present	Present	Present

The genetic basis of HSD/hEDS is currently unknown, as there are gene discovery projects underway, to accommodate future identification of disease-causing genes, it was agreed a diagnosis of hEDS can be made in a child of a parent with hEDS and a confirmed new genetic cause if the child has both the disease-causing mutation and GJH.

### Age range in this framework

The agreed minimum age at which GJH should be assessed, including comparison to age and sex specific reference data, was 5 years old as infants and toddlers have insufficient bony maturity for clinically meaningful assessment [4]. It is also applicable to all adolescents who are still maturing biologically. Biological maturity is defined as skeletal maturity with growth velocity less than 1 cm/year using 2 measures at least 3 months apart, or a mature bone age x-ray [13]. Adolescents who reach biological maturity before 18 years old, and young adults aged 18 years and over should be reassessed against the current 2017 criteria [1].

### Beighton score and GJH

In children, GJH is currently established at a cut-off Beighton score of  ≥ 6/9. Recent research has reported divergence between sexes at a statistically significant level present in 14-year-olds [3] and occurring in 14–19-year olds [4]. Neither study provided enough information about biological maturity to assess the relevance of these findings to biologically immature adolescents, so the cut off at 6/9 was retained.

Inter-rater and intra-rater reliability of the Beighton score has been found to be substantial to excellent [14] and is well-known, quick, and easy to use. However, more detailed assessment of GJH and joint laxity are recommended for planning clinical interventions and future research on phenotyping. In particular, the Upper Limb Hypermobility Assessment Tool (ULHAT) [15] (yet to be validated in children) and Lower Limb Assessment Score (LLAS) [16]. Both are 12-item tests assessing joints in multiple planes of movement.

While a Beighton score of  ≥ 6 is used in this framework, a comprehensive musculoskeletal examination should always be undertaken and include neurological, neuromuscular, and skeletal assessment, and a detailed assessment of each symptomatic joint.

### Skin and other connective tissue abnormalities

The group consensus was to retain the 5 skin items in alignment with the 2017 hEDS adult criteria despite “unusually soft skin” and “mild skin extensibility” being largely subjective and no data being available on piezogenic papules in children. In terms of outcomes, the presence of scarring and stretch marks predicted greater disability at 3 years whereas soft skin and stretchy skin did not [17].

It was agreed that at least any 3 of 5 of the skin features need to be present to consider skin involved. As many young children have not sustained injuries resulting in significant scarring the requirement for 2 atrophic scars was agreed to be reduced to one. Clinically, unexplained striae would be an unusual finding prior to adolescence.

Congenital umbilical hernia is a common phenomenon in the pediatric population and inguinal and umbilical hernia are common post neonatal intensive care, so hernias of this nature should not be counted. However, recurrent hernias are unusual and suggest a degree of tissue fragility. A recent case control study in 3–10-year-old children presenting with inguinal hernia found a statistically significantly higher proportion of patients with GJH at Beighton score ≥ 6/9 in children compared to age and sex matched controls [18].

### Musculoskeletal (MSK) episodic, activity-related pain

The association between GJH and acute onset intermittent MSK pain has been found in several population studies of adolescents [19, 20], and one study in symptomatic children [21]. Assessment of a painful joint(s) for presence of instability and potentially related soft tissue injury may identify a treatable cause. In the Sydney clinical cohort of 89 patients aged 6–16 years, with Beighton scores ≥ 4 at inception, the commonest complaint was joint pain with 94% reporting pain in multiple joints (mean 6.4 joints, range 0–15), at the knee 63%, foot 50%, ankle 48%, hand 36%, wrist 33%, back 27%, hip 26%, shoulder 22%, and elbow 21% [22]. Chronic widespread musculoskeletal pain is considered a comorbidity and discussed below.

### Joint instability

Overall, within the general population, adolescents have a notably higher incidence of initial and recurrent patella dislocation compared to adults [23]. In the Sydney cohort [22] patients commonly reported joint instability (67% overall) at the following sites knee (predominantly patella) (35%), ankle (32%), shoulder (26%), hand (18%), and wrist (14%). Mixed pediatric and adult studies support these findings. In the knee, Nomura et al. [24] found patients with recurrent patella instability had higher rates of GJH than controls (24% vs. 10% based on Carter Wilkinson ≥ 4). A recent systematic review also found that young athletes with joint hypermobility were three times more likely to have shoulder injuries e.g., dislocation, compared with athletes without joint hypermobility (OR = 3.25, *p* = 0.001) [25].

### Soft tissue injuries

There is high quality evidence of an increased risk of soft tissue injuries in adolescents with identified EDS or joint hypermobility evolving from mixed pediatric and adult studies. In a Danish population registry study (n = 48,019, median age 31 IQR 16–43) patients presenting at hospital with all types of EDS (ICD10 Q79.6, n = 1319) and not including GJH/HSD had musculoskeletal contusions and sprains at more than 10% frequency. Meniscal injuries and “unspecified” knee injuries were commoner in people with EDS than controls [26].

Further, hypermobile sporting participants (age range 9–39 years, Beighton score ≥ 7), have an increased risk of knee joint injuries compared to those with a Beighton score 4–6 [27]. While a prospective study of children aged 9–14 years did not demonstrate an increased risk of injury it appears underpowered with only 36 of the 999 children classified as hypermobile (Beighton score ≥ 5–9) [28]. The group consensus was that for clarity and consistency the need for surgery, or imaging findings of tissue damage was the minimum required to provide certainty of soft tissue injury and a definitive diagnosis of a local cause.

### Core comorbid conditions

The group agreed to include comorbid conditions which have been reported in at least cohort studies, and at frequencies of more than 10% [22]. A fundamental challenge is that comorbidities have multiple potential associations and are not independent of each other; for example, widespread musculoskeletal pain and dysautonomia are both common comorbidities of chronic fatigue (Table 2). The group considered comorbidities to add complexity to an individual's presentation, so although they do not influence the diagnosis overall, a different subtype in the framework is assigned if they are present and distressing or disabling to the child.

**Table 2:** Complexity, the relationship between comorbidities and each other, irrespective of joint hypermobility

Comorbid symptom	MSK pain	Fatigue	FGID	Bladder dysfunction [29]	Orthostatic intolerance or POTS	Anxiety
Chronic pain [30]		59% (53)	41% appetite disturbance (53)	23–27% (52)		> 50% (53)
Chronic fatigue [31]	Common symptom		Nausea common symptom	20x increase	Secondary diagnosis	29% [32]
FGID [33]	24%	94%		35%	94% dizziness, 24% tilt table confirmed PoTS	
Bladder dysfunction	9–12%	2× increase	7–48%			
Primary dysautonomia [34]	Joint pain > 40%, muscle pain > 30%	> 90%	Nausea, early satiety > 60%, abdominal pain > 50%, constipation diarrhea and vomiting all > 20%	68% (note adults only) [35]		
Anxiety [36]	45% muscle tension	35%	70% stomach ache, 17% swallowing difficulties	25% urge incontinence	25% dizziness	

This table summarises rates of each comorbidity in populations with another comorbidity, so reading across, in a population with chronic fatigue 20% have anxiety and in a population with anxiety 35% have fatigue symptoms

The core comorbidities identified have established diagnostic definitions, and include chronic primary pain by the ICD-11 [37], CDC Institute of Medicine persistent fatigue [31], Rome Foundation Rome IV Criteria for functional gastrointestinal (GI) disorders [38], functional bladder disorders described by the NIH National Institute of Diabetes and Digestive and Kidney Diseases (NIH NIDDK) [39], dysautonomia defined by the NIH National Institute of Neurological Disorders and Stroke (NIH NINDS) primary dysautonomia [40], and the American Psychiatry Association DSM 5 for anxiety [41]. The consensus was that the current diagnostic frameworks for each of these conditions should be used in an individual who also has hypermobility.

#### Chronic pain

Chronic pain, defined using the WHO ICD-11 [37], is pain that persists or recurs for longer than 3 months and is associated with significant emotional distress and/or functional disability. In the WHO ICD-11 framework chronic musculoskeletal pain is considered secondary to Ehlers–Danlos syndrome. In clinical practice chronic visceral, headache, and orofacial pain can also be encountered in this group so the consensus was that chronic pain in general should be considered a core comorbidity.

In a general population cohort [19] participants at 17.8 years who had a Beighton score ≥ 6–9 at 13.8 years demonstrated a weak suggestion of a higher risk of chronic regional pain and chronic widespread pain (OR 1.84 (CI 0.97–3.50, *p* = 0.062) and 2.27 (CI 1.22–4.22, *p* = 0.01) respectively). Significantly increased rates of extremity pain and abdominal pain were reported in patients across the lifespan with EDS (ICD-10 DQ796) (n = 1319) compared to database controls (n = 46,700); about 400 of the EDS patients were diagnosed in childhood [26]. Pain-related disability was found in the Sydney cohort [17] as well as 47 10–20-year-olds with hypermobility (Beighton score 4–9). Children and adolescents in these studies also reported headache [42], and recurrent abdominal pain [43].

There may be involvement of the central nervous system in the development of chronic musculoskeletal pain in this group, demonstrated by low pressure pain thresholds [44].

#### Chronic fatigue

Fatigue diagnosed per the 2015 CDC IOM criteria [31], after other medical causes have been excluded, can present in children with hypermobility. In a cohort of 47 10–20-year old's (Beighton score 4–9) self-reported fatigue was greater than controls, with higher fatigue-related disability [42]. Worse fatigue correlated with more functional impairment at 3 years in the prospective Sydney cohort [17] and worse quality of life at inception [43]. The mechanisms underlying chronic fatigue in GJH are yet to be determined.

#### Functional gastrointestinal disorders (FGID)

Functional gastrointestinal disorders (FGID) can present in pediatric patients with hypermobility. These conditions are diagnosed using the Rome criteria [38]. Constipation is the commonest symptom, followed by diarrhea and recurrent abdominal pain, with 54% of the Sydney cohort reporting any GI symptom and 17% reporting more than one [22]. Clinically, upper GI symptoms such as nausea and early satiety present more commonly in adolescents. The diagnosis of FGID is made once other gastrointestinal disorders such as celiac disease and inflammatory bowel disease have been excluded. Pediatric studies of populations of children with FGID using the Beighton score ≥ 4 cut off in the literature [33, 45–47] have been negative or uncontrolled. No studies have reported the prevalence of FGID in hypermobile children in comparison to controls. However, significantly increased rates of FGID were reported in a lifespan registry cohort, in 13% of the EDS population compared to 3.9% of controls (*p* =  < 0.001) [26]. The group consensus was these conditions were important comorbidities. The presence of diarrhea at baseline in the Sydney cohort was significantly more common in the children with severe functional disability compared to those with milder functional disability [17]. There is weak to very weak evidence for possible mechanistic links with local connective tissue, anatomical, and physiological abnormalities.

#### Functional bladder disorders

There is increasing evidence for the presence of lower urinary tract dysfunction, defined using the NDDIK criteria [39], as part of the clinical presentation of symptomatic hypermobility, and the group agreed it was an important comorbidity. Several studies have identified an increased prevalence of urinary incontinence in hypermobile pediatric case-controlled studies [22, 48, 49]. The prevalence of symptoms of stress incontinence in the Sydney cohort was 26% and this predicted reduced quality of life in hypermobile children [22]. It has been suggested that abnormalities in collagen may cause a dyssynergy of the pelvic floor musculature [50].

#### Dysautonomia

There is growing recognition of an association between autonomic dysfunction and hEDS/HSD and the group agreed it is an important comorbidity, commonly presenting as Postural Orthostatic Tachycardia Syndrome (POTS) in older children. These disorders are defined by the NINDS framework [40]. In a pediatric POTS registry, 62% of patients had hEDS/HSD (Beighton score ≥ 5/9), and earlier median age at symptom onset (12.1 vs. 13.5 years, *p* = 0.004) and longer median symptom duration (2.5 vs. 1.5 years, *p* = 0.0008) than patients without hypermobility [34]. The Sydney cohort reported orthostatic intolerance symptoms in 39% of the group [22]. Adolescents and young adults with G-HSD and hEDS demonstrate worse symptoms and more disability associated with orthostatic intolerance than their non-hypermobile age and gender matched peers [51].

#### Anxiety

Anxiety, diagnosed using DSM-5-TR criteria [41], was determined to be an important comorbidity by the group. A Welsh population registry study found an increased frequency of coded “mental disorders” in children (under 18) with EDS/hEDS/HSD, with an odds ratio of 4.16 (95% confidence intervals 3.29–5.27) [52]. Bulbena-Cabre et al. found that 160 children with GJH age 5–17 (Beighton score ≥ 4) had significantly higher rates of anxiety disorders (*p* = 0.035) compared to a control group [53]. In 93 children aged 8–15 years, with anxiety the prevalence of GJH (Beighton score ≥ 6) was three times higher than controls [54]. The presence of psychiatric disorders was found to be associated with a lower quality of life score in 47 children and adults (ages 10–20 years) with hEDS/HSD (Beighton score 4–9) [42]. In a study of older adolescents and adults (n = 168) further association between GJH and anxiety was affirmed [55]. One systematic review including adolescents and adults (range 12–48 years, Beighton score ≥ 4) demonstrated a significantly greater probability of anxiety, depression, and panic disorders than unaffected controls [56].

Overall, the evidence is moderate in quality for anxiety and emerging for other mental health disorders, so the consensus decision was to include anxiety only at this stage. The underlying mechanisms are currently unknown.

#### Emerging comorbidities

The group recognized that there is potentially association of other diagnoses and hypermobility, listed in Table 3. These were not included as group consensus was the comorbidity was likely to be low frequency (less than 10%), or the evidence of association was currently weak.

**Table 3:** Emerging comorbidities in Children and Adolescents with HSD and hEDS

Comorbidity	Description	Study
Inflammatory arthritis	The term juvenile episodic arthritis/arthralgia for patients with hypermobility and recurrent symptoms of arthritis was introduced in 1985 and further described in 2005 and 2017. No detailed epidemiology is available.	Gedalia et al. [57] Adib et al. [58] Cecchin et al. [59] Pessler et al. [60] Farrokhi et al. [61]
Developmental coordination disorder (DCD)	Neurodevelopmental problems, GJH and HSD are a growing area of interest, as they might explain a subgroup of hypermobile people who struggle with body awareness. Kirby and Davies reported the prevalence of Joint Hypermobility Syndrome (JHS) in children with DCD was 37% versus typically developing children (7.4%) (*p* < 0.05).	Kirby and Davis [62]
Attention deficit hyperactivity disorder (ADHD)	Nair et al. showed ADHD affects 5% of school-aged children and often occurs before age 4 (Nair et al., 2006). Dogan et al. showed that the prevalence of JHS measured by a Beighton score ≥ 4 in 54 children (mean age of 9.7) with ADHD was 31.5% compared to 13.9% of non-ADHD controls (*p* = 0.05). In a case control study, Shiari et al. found the prevalence of joint hypermobility (Beighton score ≥ 4) was 74.4% in 86 Iranian children with ADHD and 12.8% in 86 healthy controls (*p* < 0.001). In a retrospective case series, Kindgren et al. reviewed data from 201 children with hypermobility disorders. Significantly more with a registered ICD-10 Q79.6 EDS diagnosis had ADHD compared to children with M35.7 hypermobility syndrome diagnosis (*p* = 0.02). 16% of children with either diagnosis had ADHD.	Nair et al. [63] Dogan et al. [64] Shiari et al. [65] Kindgren et al. [66]
Autism spectrum disorder (ASD)	A systematic study by Shetreat et al. found that children with ASD had significantly greater joint mobility (*p* < .002), more gait abnormalities (*p* < .0001), and on average walked 1.6 months later than their non-autistic peers. The etiological mechanisms underlying the comorbidity between ASD and HSDs are emerging. Eccles et al. reported structural brain differences between subjects with and without JH in areas involved in emotion processing, attention, cognitive control of pain, and negative emotions which may be relevant.	Shetreat et al. [67] Eccles et al. [68]
Menorrhagia and dysmenorrhea	Hickey reported 7/13 (54%) of a case series of younger patients (9–18 years old) with hEDS per Villefranche criteria with menorrhagia post menarche. Menorrhagia and dysmenorrhea above normal for the general population are potentially an issue for older adolescent females.	Hickey [69]
Adolescent idiopathic scoliosis (AIS)	Czaprowski found statistically significantly higher prevalence of GJH (defined as Beighton score 4 or more and 2 positive Hakim and Grahame 5-part questionnaire responses) in patients with AIS than controls. A recent systematic review found no association, the “wide variation in methods of musculoskeletal hypermobility” made study comparisons difficult so it was somewhat inconclusive. Large studies of an unselected population with both well-defined AIS and well-defined hypermobility are needed to answer this question.	Czaprowski et al. [70] Shere and Clark [71]

## Conclusion

We present an updated diagnostic framework for pediatric patients with generalized joint hypermobility and associated conditions, to be used before biological maturity and after other diagnoses have been actively excluded. This is a key resource for clinicians and researchers to ensure consistency in diagnosis and in accurately identifying pediatric clinical and research cohorts. Future work is needed to explore the broad differential diagnosis for this group, management of the condition, and test this framework. Prospective cohort studies of the evolution of this phenotype and its relationship with adult hEDS would be of particular interest.

## Data Availability

Data sharing is not applicable to this article as no datasets were generated or analysed during the current study.

## Abbreviations

CDC: Centers for Disease Control and Prevention
FGID: Functional gastrointestinal disorders
GI: Gastrointestinal
GJH: Generalized joint hypermobility
ICD: International classification of diseases
EDS: Ehlers–Danlos syndromes
hEDS: Hypermobile Ehlers–Danlos syndrome
HSD: Hypermobility spectrum disorders
NIH: National Institute for Health
NINDS: National Institute of Neurological Disorders and Stroke
NIDDK: National Institute of Diabetes and Digestive and Kidney Diseases
pGJH: Pediatric joint hypermobility
pgGJH: Pediatric generalized joint hypermobility
POTS: Postural orthostatic tachycardia syndrome
WHO: World Health Organisation
